# Retraction: LncRNA AWPPH promotes the growth of triple-negative breast cancer by up-regulating frizzled homolog 7 (FZD7)

**DOI:** 10.1042/BSR-2018-1223_RET

**Published:** 2022-05-10

**Keywords:** AWPPH, frizzled homolog 7, LncRNA, proliferation, Triple-negative breast cancer

This article is being retracted from *Bioscience Reports* at the request of the authors. Further experiments performed by the authors showed that LncRNA AWPPH had no promotion of cell growth in HCC38, HCC1187 or 4T1. Thus, the conclusion that LncRNA AWPPH is a tumor promoter in TNBC should be further studied to validate the results.

The Editor-in-Chief and Editorial Board agree with the retraction.

